# Sustainable LC3 Concrete in the Circular Economy: Assessment of Mechanical, Microstructural, and Durability Characteristics with Surkhi, Metakaolin, Nano‐Silica, and M‐Sand Blended Concrete

**DOI:** 10.1002/gch2.202500026

**Published:** 2025-04-10

**Authors:** Badrinarayan Rath, Praveenkumar T R, Kavindra Singh Dhami, Prabhu Paramasivam, Mohamed Yusuf

**Affiliations:** ^1^ Department of Civil Engineering Graphic Era Deemed to be University Dehradun Uttarakhand 248002 India; ^2^ Department of Research and Innovation Saveetha School of Engineering, SIMATS Chennai Tamil Nadu 602105 India; ^3^ Department of Peace and Development Studies Njala University BO Campus –18 Sierra Leone

**Keywords:** LC3 concrete, microstructural improvement and sustainability, M‐sand, nano silica, Surkhi

## Abstract

Researchers are increasingly focused on eco‐friendly concrete with reduced carbon footprints. Among sustainable options, Limestone Calcined Clay Cement (LC3) concrete offers enhanced strength and durability with lower greenhouse gas emissions. This study evaluates the mechanical, microstructural, and durability characteristics of LC3 concrete modified with surkhi and nano‐silica as cementitious materials, replacing metakaolin and gypsum. Surkhi and nano‐silica are varied from 0%–40% and 0%–4%, respectively, while fine aggregate is completely replaced with M‐sand to improve packing density. Ten M30‐grade concrete mixes are analyzed after 28 and 90 days of curing. By incorporating surkhi and nano‐silica as partial replacements for metakaolin and gypsum in LC3 concrete, the research investigates potential improvements in strength, durability, and microstructural integrity of the concrete and provides lower greenhouse gas emissions compared to traditional Portland cement. Results revealed that surkhi and nano‐silica significantly improved strength and microstructure, with surkhi optimally limited to 30%. M‐sand proved effective in enhancing durability against weathering. These findings position modified LC3 concrete as a sustainable alternative, offering improved performance and advancing its potential within the circular economy framework.

## Graphical abstract

Eco‐Friendly LC3 Concrete – Enhanced with surkhi, nano‐silica, and M‐sand. Performance Boost – Improved strength, durability, and microstructure. Sustainability Impact – Lower emissions and circular economy benefits.

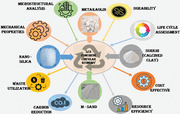

## 1 Introduction

Cement is most widely used construction material in construction industry globally, considered as corner stone of modern infrastructure.^[^ ^1^ ^]^ The extensive use of concrete comes with significant environmental cost due to high carbon footprint of cement production and it contributes almost 8% of global carbon dioxide emissions.^[^ ^2^ ^]^ The significant need for sustainable development has driven researchers to explore innovative materials and methodologies that can effectively mitigate the environmental impact without affecting the performance of concrete,^[^ ^3^, ^4^ ^]^ Currently, the global construction industry is facing dual challenges; meet the growing demand for infrastructure and address the environmental concerns due to cement production.^[^ ^5^ ^]^ Cement, considered to be primary binder in concrete plays a vital role in construction industry and is considered as energy intensive. The substantial release of greenhouse gases during the calcination of limestone and subsequent clinker production is compelling the industry to seek for sustainable alternatives,^[^ ^6^, ^7^ ^]^ Supplementary cementitious materials include metakaolin and nano silica offers a promising solution for this environmental concern. Replacement of supplementary cementitious materials with cement not only reduces the carbon footprint and also enhances the mechanical and durability properties of concrete.^[^ ^8^, ^9^, ^10^ ^]^

Among various supplementary cementitious materials, metakaolin is considered to be highly reactive pozzolanic material, mainly derived from thermal activation of kaolinite clay.^[^ ^11^ ^]^ When metakaolin reacts with portlandite, byproduct of cement hydration forms an additional calcium silicate hydrate gel, which improves the compressive strength, impermeability, and durability of concrete,^[^ ^12^, ^13^, ^14^ ^]^ Due to this reaction mechanism, it is considered as attractive choice for high performance applications. The high surface area and silica content of metakaolin facilitates the concrete matrix densification and reduces the voids which enhances the overall structural integrity of concrete specimens.^[^ ^15^ ^]^ LC3 concrete is blend of clinker, limestone and calcined clay and serves as an ideal platform integrating advanced SCMs like metakaolin and nano silica. These materials not only reduce the environmental footprint and also align with LC3's goal of reducing cement clinker content in addition to improvement of mechanical and durability characteristics.

Multifaceted benefits of incorporating metakaolin in concrete have been demonstrated by several researchers. Research on calcined clays derived from high‐ and low‐grade kaolinite shows superior performance in enhancing chloride resistance and electrical resistivity. Low grade calcined clays showed lower efficiency as compared with high grade metakaolin, but still the results of low‐grade calcined clay outperformed conventional fly‐ash based concrete in terms of durability properties.^[^ ^16^ ^]^ Utilization of metakaolin showed notable improvements in white concrete, where synergetic blend of 15% metakaolin, 10% calcium carbonate for replacement of white cement enhances the compressive strength, reduces the carbon emissions and refines the microstructure which contributes to sustainable architectural applications.^[^ ^17^ ^]^ The durability and mechanical properties of recycled aggregate concrete have been significantly improved through incorporation of metakaolin. Refinement of microstructure due to incorporation of metakaolin showed improved resistance to sulfate induced expansive products, enhances peak stress and elastic modulus under dynamic conditions.^[^ ^18^ ^]^ The industrial biproducts like fly ash and pond ash increased the durability property of concrete.^[^ ^19^ ^]^ In addition, incorporation of metakaolin with 10% fly ash and silica fume blended samples enhances acid resistance, sorptivity and mechanical strength, especially in recycled aggregate concrete with 50% aggregate replacement level.^[^ ^20^ ^]^ Inclusion of metakaolin along with recycled concrete powder (RCP) reduces the water absorption and void index. Mechanical properties of RCP and metakaolin blended specimens showed enhanced performance over period. This dual approach optimizes the physical and mechanical characteristics of mortar and reduces the dependence of traditional cement.^[^ ^21^ ^]^ Additionally, metakaolin combined with 15% fly ash in recycled fine aggregate concrete (RFAC) showed improved compressive strength and carbonation resistance, with optimal performance observed at a 60% replacement level of natural aggregates.^[^ ^22^ ^]^

Nano silica, characterized by high surface area and ultrafine particle size revolutionized the concept of particle packing in concrete technology,^[^ ^23^, ^24^ ^]^ It acts as both filler material and pozzolanic material, bridges the gap at the micro and nano levels.^[^ ^25^ ^]^ Due to this pozzolanic reaction, it significantly reduces the porosity. The spherical morphology and high reactivity of nano silica accelerates the cement hydration and pozzolanic reactions, produces more CSH gel and improves the microstructure of concrete matrix,^[^ ^26^, ^27^ ^]^ The incorporation of nano silica in concrete specimens not only improves the mechanical properties such as compressive and tensile strength, but also shows superior performance against chemical attacks, making it ideal to consider in extreme environmental conditions.^[^ ^28^, ^29^ ^]^

The scarcity and environmental degradation associated with natural sand extraction necessitated the researchers to adopt alternatives to fine aggregates. Natural sand, generally sourced from river beds poses ecological risks such as habitat destruction and water depletion.^[^ ^30^ ^]^ Manufactured sand produced by crushing hard granite can be considered as viable substitute for conventional river sand due to its controlled particle size distribution and angular shape,^[^ ^31^, ^32^ ^]^ The rough texture and angularity of M‐sand might reduce the workability, but these characteristics improves the interfacial bonding between cement paste and aggregate which leads to superior mechanical properties.^[^ ^33^ ^]^ The combined blend of M‐sand and supplementary cementitious materials has shown significant potential to address both sustainability and performance challenges,^[^ ^34^, ^35^ ^]^ Despite the advancements, the combined effect of metakaolin, nano‐silica and M‐sand on concrete strength, workability and durability remain unexplored. Existing researchers mainly focuses on binary blends and it leaves the critical gap to explore and understand the behavior of ternary interactions. In addition, the optimal performance of these materials to achieve the balance between sustainability and performance were not fully established.

The present study investigates the engineering properties of concrete incorporating lime, metakaolin, Gypsum, surkhi, nano‐silica and M‐sand as partial replacements for cement and natural sand. The research aims to develop sustainable concrete mixes to reduce cement consumption and utilize alternative materials without compromising the mechanical and microstructural characteristics. This study examines the effect of flowability, compressive strength, split tensile strength, flexural strength, and microstructural characteristics of metakaolin and nano‐silica blended concrete specimens with replacement level of nano silica ranges from 2% to 4% and metakaolin from 10% to 40%. The environment sustainability of the mix can be assessed and enhanced by replacing natural sand with complete replacement of M‐sand.

## 2 Research Significance

**Figure 1:**
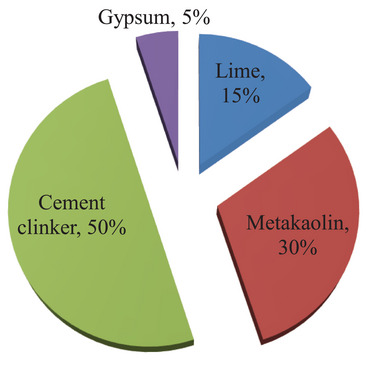
Compositions of LC3 concrete with their percentages which are used by previous researchers.^[^ ^38^, ^39^ ^]^

Cement production poses a significant threat to environmental sustainability due to the substantial release of greenhouse gases and high energy consumption. The extensive use of cement directly and indirectly contributes to environmental pollution. To mitigate cement consumption, the exploration of alternative supplementary cementitious materials is essential. Limestone calcined clay cement (LC3) has emerged as a sustainable and innovative alternative to traditional cement. The composition of LC3 is shown in **Figure** **1**. By leveraging abundant materials such as limestone and low‐grade clays, LC3 can reduce CO₂ emissions by up to 40%. It is also cost‐effective and compatible with existing cement plants, requiring no significant capital‐intensive modifications. The primary components of LC3 include clinker, calcined clay, limestone, and gypsum. Calcined clay is produced by heating clay to high temperatures, which enhances its properties and makes it suitable for various applications. Typically, metakaolin is used in LC3 concrete as a cacined clay. Another form of calcined clay, known as surkhi, is made by grinding burnt bricks or brick‐bats into a fine powder. These brick‐bat fragments, often considered waste products at brick kilns in the Indian subcontinent, can be easily processed into surkhi for use in LC3 production. Hence surkhi can be used as a partial replacement of metakaolin. Similarly, nano silica, derived from agricultural, electronic, and industrial by‐products, also serves as a potential substitute. In LC3, gypsum has a crucial role in controlling the mechanical, workability, and hydration characteristics.^[^ ^36^ ^]^ Being nano silica is a finer particle and spherical in shape,^[^ ^37^ ^]^ it acts as ball bearing action in between the cementitious materials, which increases the workability of concrete. Hence, the gypsum can be partially replaced by nano silica in LC3 cement. Meanwhile, natural sand scarcity, caused by illegal mining and seasonal challenges such as difficulty in sourcing during the rainy season, necessitates alternative filler materials. Manufactured sand (M‐sand) serves as a viable substitute for natural sand in concrete production.

In this research, a new type of LC3 concrete was developed by partially replacing metakaolin with surkhi and gypsum with nano silica. M‐sand was used as a filler material instead of natural sand. M30 grade concrete was prepared using various combinations of cement, metakaolin, surkhi, gypsum, nano silica, M‐sand, and coarse aggregate. Concrete specimens, including cubes, cylinders, and beams, were cast to evaluate properties such as flowability, microstructural characteristics, and static mechanical performance.

Limited research has been conducted on flowability and microstructural analysis using the binary replacement in LC3 cement, surkhi, and nano silica as a binder, along with M‐sand as a filler material in LC3 concrete. The proposed mix design offers the potential to reduce the cement consumption in LC3 concrete, thereby promoting environmental sustainability and contributing to a cleaner and greener future.

## 3 Materials and Methodology

**Figure 2:**
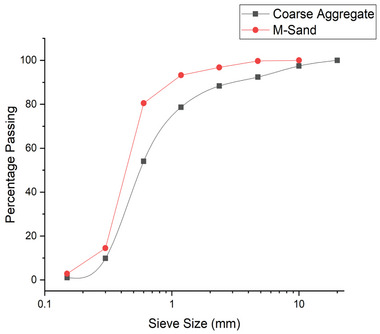
Particle size distribution for M‐sand and coarse aggregate.

The study integrates various constituents from different sources, utilizing surkhi and nano silica as replacement materials of metakaolin and gypsum in LC3 cement and M‐sand as a filler material in LC3 concrete. The physical and chemical properties of cement, nano silica, and metakaolin are presented in **Table** **1**. M‐sand, conforming to IS: 383‐1970 [Bureau of Indian Standards, Manak Bhavan, 9 Bahadur Shah Zafar Marg, New Delhi, 110002, India] grading zone III, was employed as the fine aggregate. It was carefully sieved using a 4.75 mm mesh to remove larger particles, followed by thorough washing to eliminate dust. Locally sourced coarse aggregates, with a maximum size of 20 mm, were tested according to IS 383–1970 standards. These aggregates were meticulously cleaned to remove residual dust and dried to a surface‐dry condition before use. The particle size distributions of M‐sand and coarse aggregates are illustrated in **Figure** **2**. The selection of component materials for conventional M30‐grade concrete was carried out following the guidelines of IS: 10262‐2019 [Bureau of Indian Standards, Manak Bhavan, 9 Bahadur Shah Zafar Marg, New Delhi, 110002, India]. In order to lower the amount of cement clinker in LC3 concrete, the composition was designed to contain 30% cement, lime, and calcined clay, with the remaining 10% being gypsum. A total of eight LC3 concrete mixes were prepared using combinations of surkhi and nano silica, along with one mix of conventional concrete for comparative analysis. surkhi and nano silica were used as partial replacements for metakaolin and gypsum. Surkhi was substituted at levels of 10%, 20%, 30%, and 40% of metakaolin, while nano silica was used as a gypsum replacement material at 2% and 4%. The mix proportions for all compositions are detailed in **Table** **2**. The initial setting time was found as unchanged by replacing gypsum partially with nano silica. The addition of nano‐silica might reduce the initial setting time of the concrete. Since the amount of nano silica was very small such type of experience didn't gain in the present research. Gypsum was typically added to cement to delay the hydration of aluminate phases, preventing flash setting. Replacing it with nano‐silica, which did not provide such retardation, might lead to a quicker setting, which can be advantageous or problematic depending on the specific application needs. The maximum amount of nano silica was taken as 4% as a partial replacement material of gypsum, which was very small. Nano‐silica particles tend to agglomerate due to their high surface energy and the Van der Waals forces between them. This agglomeration could lead to uneven distribution in the concrete mix, potentially causing weak spots and reducing the overall effectiveness of nano‐silica in improving the concrete properties. Though the nano‐silica was a little bit more expensive than traditional cementitious materials due to its production and processing costs, that much cost difference had not found in this research due to the smaller replacement. The overall cost of the concrete was found as same after replacing gypsum with nano silica up to 4%.

**Table 1:** Physical and chemical properties of cement, lime, metakaolin, gypsum, surkhi, and nano siica.

Parameters	Cement	Lime	Metakaolin	Gypsum	Surkhi	Nano silica
Physical Properties						
Color	Greenish gray	White	Light gray	White	Reddish brown	White
Specific Gravity	3.15	3.2	2.6	2.4	2.6	1.2
Particle size	Fineness of 8%	Fineness of 5%	1‐2 µm	Less than 90 µm	Less than 75 µm	10 nm
Chemical Properties						
CaO	67%	95%	–	–	7%	–
SiO_2_	17%	1.5%	55%	3%	50%	99%
Al_2_O_3_	5%	0.5%	40%	0.5%	20%	–
Fe_2_O_3_	2%		1%	0.1%	7%	–
MgO	2%	2%	–	–	5%	–
SO_3_	1.5%	–	–	–	2%	–
TiO_2_	–	–	2%	–	–	–
CaSO_4_, 2H_2_O	–	–	–	95%	–	–
Other Alkalies	5.5%	1%	2%	1.4%	9%	1%

**Table 2:** Mix proportion for concrete specimen.

Mix ID	Cement [kg m^−3^]	Lime [kg m^−3^]	Metakaolin [kg m^−3^]	Gypsum [kg m^−3^]	Surkhi [kg m^−3^]	Nano Silica [kg m^−3^]	Fine Aggregate [kg m^−3^]	Coarse Aggregate [kg m^−3^]	Water [kg m^−3^]	Super plasticizer [kg m^−3^]
CM	425	0	0	0	0	0	646	1095	191	2.12
C_0‐0_	127.5	127.5	127.5	42.5	0	0	646	1095	191	2.12
C_10‐2_	127.5	127.5	114.75	41.65	12.75	0.85	646	1095	191	2.12
C_10‐4_	127.5	127.5	114.75	40.8	12.75	1.7	646	1095	191	2.12
C_20‐2_	127.5	127.5	102.0	41.65	25.5	0.85	646	1095	191	2.12
C_20‐4_	127.5	127.5	102.0	40.8	25.5	1.7	646	1095	191	2.12
C_30‐2_	127.5	127.5	89.25	41.65	38.25	0.85	646	1095	191	2.12
C_30‐4_	127.5	127.5	89.25	40.8	38.25	1.7	646	1095	191	2.12
C_40‐2_	127.5	127.5	76.5	41.65	51	0.85	646	1095	191	2.12
C_40‐4_	127.5	127.5	76.5	40.8	51	1.7	646	1095	191	2.12

The workability of the concrete mixes was assessed using the flowability test. Subsequently, 150 mm × 150 mm concrete cube specimens and 100 mm × 100 mm × 500 mm beams were cast for getting compressive strength and flexural strength of new concrete mix. After 24 h, the cube samples and beam samples were demolded and cured in water for 28 days to ensure proper hydration and strength development.

The fineness of cement or lime refers to the particle size and the total surface area of the cement or lime which is quoted in Table 2. It is a measure of how finely the particles are ground. Since the particles of cement and lime were fine, a 90‐micron sieve was typically employed to assess the fineness by determining the percentage of cement or lime particles that are retained on the sieve. This percentage indicated the material's fineness. It was found that the cement was finer than the lime.

## 4 Flowability Test

The flowability of the various concrete mixtures was evaluated in compliance with the specifications set out in the IS: 5512‐1983 [Bureau of Indian Standards, Manak Bhavan, 9 Bahadur Shah Zafar Marg, New Delhi, 110002, India]. The apparatus featured a flow table, 76 cm in diameter, marked with concentric circles for precise measurement. A specially designed mould, crafted from smooth metal in the shape of a truncated cone, was used. The mould had a base diameter of 25 cm, an upper surface diameter of 17 cm, and stands 12 cm tall. To begin, the table was cleaned thoroughly, and the mould was placed firmly at its center. The mould was then filled with mortar in two layers, each compacted with 25 strokes of a tamping rod (1.6 cm in diameter and 61 cm long, rounded at the lower end) to ensure consistency. After leveling the top layer, any excess concrete overflowing the mould was carefully removed. The mould was gently lifted upward, allowing the mortar to stand independently. Next, the table was raised and dropped 12.5 mm, repeating this action 15 times over 15 s. Finally, the spread diameter of the concrete was measured in six different directions to the nearest 5 mm, and the average spread was recorded.

## 5 Microstructure Analysis

To analyze the surface morphology, crack propagation, and failure patterns of the damaged concrete samples, Scanning Electron Microscopy (SEM) was conducted using a field emission SEM (model SEM4000) with a magnification range of 1× to 1 000 000×. Following SEM analysis, the samples were placed in a vacuum desiccator for 48 h, mounted on aluminum stubs, and gold‐coated to prevent electron charging. For SEM testing, the damaged samples were divided into marginal pieces, and chunks measuring 1 cm × 1 cm were prepared for each sample. The top surface of each chunk was carefully filed to expose the interfacial transition zone (ITZ) layer. Additionally, mortar samples incorporating up to 40% Metakaolin (MK) and Nano‐Silica (NS) at concentrations of 2% and 4% underwent Thermogravimetric Analysis (TGA). This technique was employed to assess the weight loss of samples subjected to thermal heating, thereby evaluating changes in material composition and thermal stability. TGA test was performed using an STA 449C Jupiter instrument, which measured the physicochemical changes in the metakaolin and nano silica based concrete systems across a temperature range of 0 to 1000 °C at a heating rate of 10 °C min^−1^ in a nitrogen‐controlled environment.

In order to precisely forecast the macroscopic behavior of concrete samples containing nano silica and recently introduced metakaolin, nanoindentation testing was carried out. The well‐established technique of nanoindentation uses indenter geometries and forces to examine the micromechanical properties of materials at the nanoscale. Samples intended for nanoindentation testing must have exceptionally flat surfaces. To prepare these samples, 28‐day‐cured specimens were precisely cored and sliced into cylindrical shapes measuring 30 mm in diameter and 10 mm in height. The prepared samples were secured in cylindrical molds and embedded in epoxy resin to enhance structural integrity during cold mounting. Once the resin gets dried, the surfaces were rubbed and smoothened using abrasive papers with increasing grit sizes (800, 1000, and 1500). All the residues were removed by treating the specimens in anhydrous ethanol in ultrasonic environment. Polishing was performed with diamond suspensions in decreasing particle sizes (10, 5, and 1 µm) to achieve a reflective surface and then samples were subjected to final ultrasonic cleaning step to guarantee thorough cleanliness.

**Figure 3:**
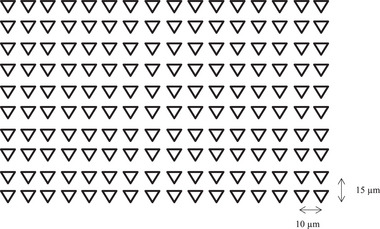
Schematic of grid indentation.

Variations in the thickness of the interfacial transition zone (ITZ) for different aggregates were observed, with the ITZ in M‐sand aggregates typically ranging from 40 to 80 µm, as reported in previous studies. Nanoindentation tests were designed to focus specifically on the ITZ between the M‐sand and the lime‐metakaolin‐surkhi‐nano silica composite binder. A grid measuring 150 µm × 90 µm was utilized for with a spacing of 10 µm horizontally and 15 µm vertically for testing as illustrated in **Figure** **3**. The measurements were conducted using an Agilent Nanoindenter equipped with a Berkovich tip. The load application followed a trapezoidal pattern, with a gradual increase at 0.2 mN s^−1^ until reaching a peak load of 2 mN, which was held constant for 10 s before unloading. The elastic modulus and hardness values were computed using standard data analysis techniques specific to nanoindentation studies.

(1) $$H=\frac{F_{max}}{A_{c}}$$

(2) $$E=\left(1-\mu^{2}\right){\left(\frac{1}{E_{r}}-\frac{1-\mu_{i}^{2}}{E_{i}}\right)}^{-1}$$

where, *F_max_* is the maximum load applied, μ is the Poisson's ratio of specimen under examination, *E_i_* is elastic modulus, *E_r_* denotes the reduced modulus, ϑ_*i*_ is the Poison's ratio specific to the diamond indenter.

### 5.1 Static Mechanical Properties Tests

In this study, samples of eco‐friendly new mixed concrete were evaluated for compressive strength, split tensile strength, and flexural strength using the IS: 516‐1959 [Bureau of Indian Standards, Manak Bhavan, 9 Bahadur Shah Zafar Marg, New Delhi, 110002, India] to assess the static mechanical properties. For compressive strength cubes of 150 mm × 150 mm × 150 mm, for split tensile strength cylinder of 150 mm and 300 mm height and for flexural strength of concrete 150 mm × 150 mm × 150 mm were cast. Concrete's flexural strength was tested on a flexural testing machine employing displacement loading at a loading rate of 0.2 mm min^−1^, while it's compressive and split tensile strengths were measured on a CTM machine. The testing apparatus recorded the greatest destructive load when the specimen failed.

## 6 Result and Discussion

Examining how surkhi and nano silica affected various concrete mixes as a binder and M‐sand affected as filler was the primary goal of the study. Investigations into the workability, strength, and microstructure of various concrete mixes were therefore carried out.

### 6.1 Flowability Test Analysis

**Figure 4:**
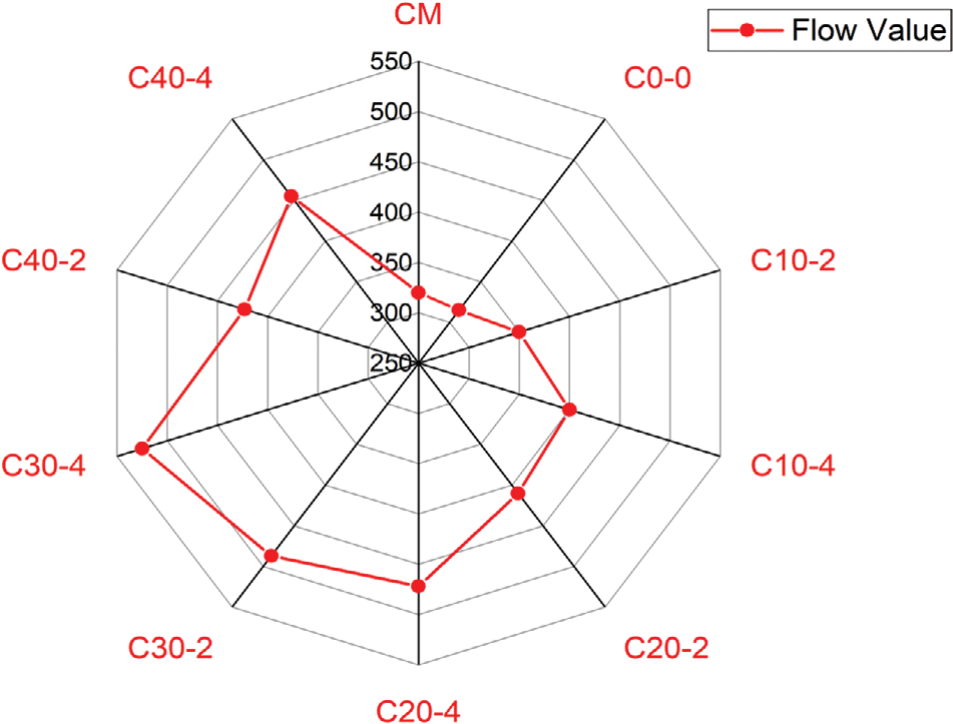
The flow value of different concrete samples against the different doses of metakaolin and nano silica used as a cement replacement material.

The test results revealed that the flow value of the traditional concrete mix was 320 mm, whereas the flow value of LC3 concrete mix was 315. The workability of concrete was decreased due to presence of calcined clay (i.e., metakaolin) in LC3 concrete, whose surface area was more. When metakaolin was partially replaced with surkhi and gypsum was partially replaced with nano silica, the flow value of the concrete increased progressively, peaking at 30% replacement with surkhi. The maximum flow value, recorded for the C_30‐4_ mix, was 525 mm, which was 1.64 times that of the traditional mix as shown in **Figure** **4**. Beyond 30% replacement, the flow value dropped to 423 mm, though it remained higher than the traditional concrete. Nano silica played a crucial role in enhancing the concrete's mobility, while the effect of surkhi varied with its dosage. At higher doses, surkhi reduced the flow value due to overfilling the voids between aggregates, which increased the concrete's porosity and reduced its workability. In contrast, nano silica, with its spherical shape, provided a ball‐bearing effect and maintained mobility without reducing the flow value. The decrease in flow value at 40% metakaolin replacement with surkhi was attributed to the formation of extra voids from excess material.^[^ ^43^ ^]^ While literature typically suggests that nano‐silica adversely impacts the flowability of composites, this study presents a contrasting observation. The exceptional smallness of nano‐silica particles in the LC3 concrete of this study allowed them to fill the gaps left by the mixture of cement, lime, and metakaolin, thus enhancing the packing density. This improved packing led to more effective water utilization; the same amount of water more efficiently wetted the surface areas of both the binding and filler particles. Consequently, the overall water demand of the concrete decreased, improving particle lubrication and enhancing flow. The tighter packing of the particles also minimized the space between them, reducing internal friction and allowing the mix to flow more freely. Therefore, at the same water‐to‐cement ratio, the mix remained more workable and was easier to handle and pour, indicating improved flowability. On the other hand, nano silica's negatively charged particles repelled each other when in contact with water, enhancing the mobility of the mix. This repulsive interaction compensated for the reduction in flow value caused by surkhi beyond the 30% threshold, ensuring a balanced performance. Again in this research M‐sand was used instead of natural sand for preparation of concrete. M‐sand might make concrete less workable, because of its rough surface roughness and angular shape, which enhanced inter‐particle friction and flow resistance. The smooth surface that allowed for simple mobility within the mix was absent from M‐sand, in contrast to the rounded particles of river sand. But the presence of nano silica might be compensated the loss of workability due to the presence of M‐sand.

### 6.2 SEM image Analysis

**Figure 5:**
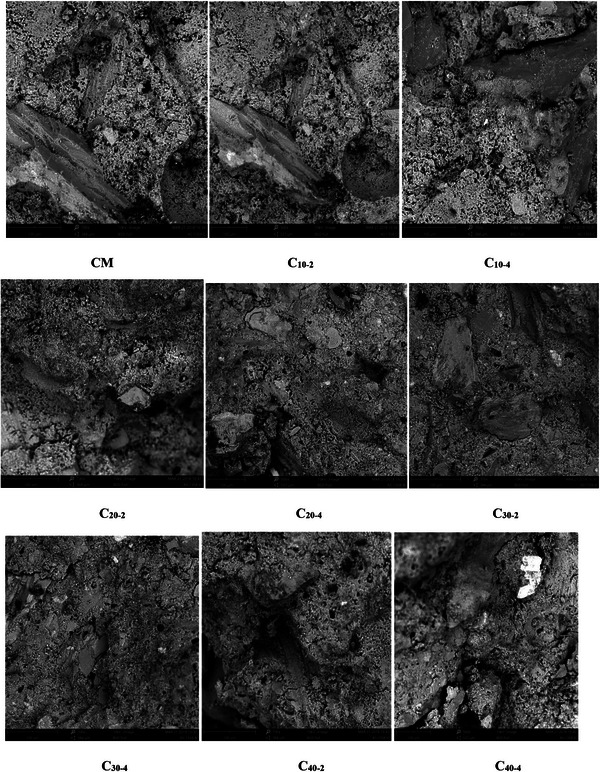
SEM images for various concrete introduced with metakaolin and nano silica.

**Figure** **5** depicts the SEM analysis of concrete samples incorporating metakaolin, surkhi, and nano silica on the microstructure of concrete mixes. The control mix shows relatively porous structure with visible hydration products CSH and Ca(OH)_2_. The presence of micro cracks and micro voids signifies the water loss during the hydration process which potentially affects the concrete matrix integrity. Incorporation of 10% surkhi and 2% nano silica in to concrete samples showed improved microstructure densification. This improvement is mainly due to increase of CSH gel formation and reduction in voids. The increase in proportion of surkhi content densifies the concrete matrix and it is observed in 20% surkhi with 2% and 4% nano silica addition (C_20‐2_,C_20‐4_) as shown in Figure 5. This improvement peaked at 30% substitution of metakaolin with surkhi, where the addition of nanoparticles in specimens further enhances the particle packing density and contributes to more compact and well‐structured matrix.

Nano silica plays vital role by acting as a filler due to its extremely fine particle size and contributes in hydration reaction mechanism to form additional CSH gel. Incorporation of 40% metakaolin makes the concrete matrix less compact with very less hydration products formation and increased porosity. This can be due to less cement content and slow reaction kinetics of metakaolin, which could not fully compensate for the hydration process. The presence of portlandite further supported the limited pozzolanic activity during the incorporation of high metakaolin proportions, which leads to compromised matrix densification. The concrete samples blended with 30% surkhi and 4% nano silica showed best microstructural properties as compared with other samples. C_30‐4_ mix showed dense and refined matrix with abundant hydration products includes CSH and CAH gels, fills the void system. The synergy between nano silica and metakaolin strengthens the concrete matrix due to presence of reactive silica and alumina. The angular particles of M‐sand further improves the interfacial transition bonding along with reduction of voids and enhancement of particle packing density. These observations confirm that optimal combination of metakaolin, surkhi and nano silica significantly improves the concrete microstructure. The higher proportion of metakaolin replacement for cement hinders the hydration reaction mechanism and compromises the matrix integrity. This finding aligns with previous studies by Fernandez et al.^[^ ^43^ ^]^ and Mukherjee and Barai^[^ ^43^ ^]^ which reported improved microstructural densification in concrete using surkhi and nano silica as supplementary cementitious materials (SCMs). Again, the densification of microstructure might be increased due to replacement of natural fine aggregate with M‐sand. This was because M‐sand's angular particles improved the bonding contact between aggregates and cementitious paste and produced a more compact arrangement. Improved mechanical qualities and durability are the outcome of the decreased porosity.

### 6.3 Analysis of Nanoindentation and Distribution of ITZ Components

Detailed nanoindentation study was performed to investigate the impact of surkhi and nano silica on the interfacial transition zone in concrete. The ITZ plays a vital role in determining the mechanical performance of concrete and it is considered to be weakest link between the aggregates and cementitious matrix. The concrete matrix composed of various hydration products include calcium‐silicate hydrate gel, usually present in low‐density and high‐density forms based on the compaction. Concrete specimens incorporating with supplementary cementitious materials and ultra‐low water‐cement ratios forms high density CSH gel, which contributes to enhancement of mechanical properties.

**Figure 6:**
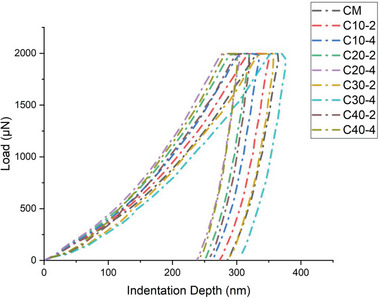
Typical P‐h curves of phases in newly proposed concrete mix.

The nanoindentation tests were performed for conventional cement concrete CM and other concrete specimens incorporated with different ratios of surkhi, metakaolin and nano silica based concrete specimens. The spacing between the indentations were maintained at 15 µm vertically and 10 µm laterally to ensure the results were not influenced by adjacent measurements. The data obtained were average of seven vertical indentations for each region, with outliers caused by voids or unhydrated particles excluded for consistency. **Figure** **6** revealed that noticeable difference was observed in ITZ characteristics between conventional concrete and blended cement concrete. Conventional concrete's ITZ showed relatively uniform modulus and hardness value than other samples and it is due to homogeneity of well hydrated cement matrix. Supplementary cementitious materials incorporated concrete showed variance in mechanical properties and it signifies the influence of its advanced composition. The modulus of elasticity and hardness of ITZ in blended cement concrete is consistently higher, ranging from 3.1% to 17.2% as compared with traditional concrete. The enhanced ITZ properties in blended concrete is mainly due to unique behavior of surkhi and nano silica. Additional C‐S‐H gel is formed in surkhi based concrete system due to presence of silica rich composition. This pozzolanic reaction releases absorbed water and the hydration efficiency were improved subsequently in addition to creation of cellular structure which fills voids and cracks. Nano silica further contributes in refining the concrete matrix and promotes the formation of densely packed hydration products. These synergetic effects significantly improve the overall strength and durability due to relatively less water absorption. C_30‐4_ concrete mix shows best performance in terms of modulus and hardness and shows greater variability in mechanical properties across interfacial transition zone. This is due to high surkhi content in cement matrix and creates slight heterogeneity in the mixture while enhancing hydration and bonding. Despite the variability, strong bond was observed between aggregate and matrix signifies less weak zones in the concrete system. The observations showed that addition of surkhi and nano silica not only strengthens the ITZ and also reduces the porosity along with improvement of mechanical performance. The unique grid structure of surkhi reacts with calcium hydroxide plays a vital role in improving cement matrix. The C_30‐4_ mixture exhibited greater variability in nano mechanical properties at the new interface, attributed to the addition of surkhi, which reduced mixture homogeneity.

Luca et al.^[^ ^43^ ^]^ study suggested that the elastic modulus of the new concrete matrix was observed to be ≈22%–26% lower as compared with old matrix, with the modulus gradually increasing as the distance from the old matrix interface increases. This finding differs from the results of the current study. The discrepancy arises from differences in material composition; previous studies focused on plain concrete where C‐S‐H phases predominate, making modulus and hardness differences largely dependent on hydration levels. In contrast, surkhi and nano silica based concrete samples featured the higher proportions of C‐S‐H phases, resulting in a more pronounced increase in modulus and hardness.

### 6.4 Thermo Gravimetric Analysis

**Figure 7:**
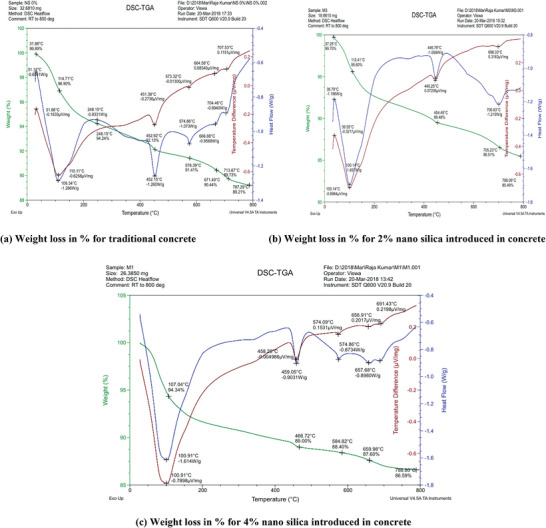
TGA curves for various mixes of concrete samples.

Thermogravimetric analysis (TGA) was conducted on various LC3 concrete samples incorporating surkhi and nano silica. The powder samples were extracted from the core of concrete cubes after 28 days of compressive strength testing. A gradual mass loss was observed in all samples as the temperature increased. This mass loss was attributed to the evaporation of moisture and chemical transformations within the LC3 concrete. As shown in **Figure** **7**, the first phenomenon observed in the TGA curve at 50, 55, and 60 °C was an exothermic peak, corresponding to mass loss in the TGA curve for traditional concrete, LC3 concrete with 2% nano silica, and 4% nano silica, respectively. This mass loss was caused by the evaporation of free and absorbed interstitial water in the samples. The second phenomenon in the TGA curve occurred at 250, 410, and 450 °C, presenting an endothermic peak associated with mass loss in the TGA curve for traditional concrete, concrete with 2% nano silica, and concrete with 4% nano silica, respectively. This mass loss was due to the release of structural water and the decomposition of hydroxyl groups from Si–O and Al–O bonds, with an approximate mass loss of 5%. These results align with those reported by Aliabdo et al.^[^ ^44^ ^]^ Overall, nano silica demonstrated a higher resistance to mass loss at elevated temperatures compared to surkhi. The mass loss decreased with increasing nano silica content. Surkhi could be incorporated into traditional concrete up to 30%. At higher temperatures, particularly 590, 650, and 670 °C for 0%, 2%, and 4% nano silica‐based concrete, mass loss was observed due to the decarbonization of by‐products such as CaCO₃ and NaCO₃. Samples cured at ambient temperature exhibited greater mass loss at shorter curing ages, which could be mitigated with extended curing periods. In this study M‐sand was introduced for increasing of the packing density. It was observed that the presence of M‐sand did not affect more on the loss of weight of the concrete.

### 6.5 Compressive Strength

**Figure 8:**
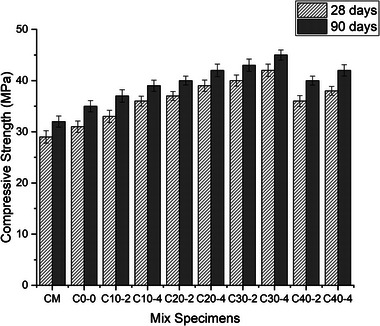
Compressive strength of various concrete samples at 28 days and 90 days curing.

**Figure** **8** illustrates the compressive strength development of the concrete mixes. As expected, compressive strength increased with age due to the progressive hydration of cement under suitable curing conditions, the partial replacement of cement with the combination of surkhi and nano silica caused an increasing of compressive strength at 28 days and 90 days. Regarding later age compressive strength, it was found that mix C_10‐2_ (10% surkhi and 2% nano silica) demonstrated a 6.4% and 5.7% higher compressive strength at 28 days and 90 days respectively compared to the compressive strength of control mix. As the replacement of metakaolin and gypsum with the surkhi and nano silica was increased the compressive strength also gradually increased. This trend continued till the percentage of metakaolin replacement reached at 30% with surkhi. This increase can be attributed to surkhi's high SiO₂ content and moderate Al₂O₃, which are key components of pozzolanic materials. As the replacement level of metakaolin with surkhi increased beyond 30%, the reduction in highly reactive cement compounds led to diminished hydration and, consequently, a decrease in compressive strength. The inclusion of nano silica significantly enhanced compressive strength. For instance, adding 4% nano silica with 10% surkhi (mix C_10‐4_) resulted in a 16.1% increase in strength at 28 days and 11.4% increase in strength at 90 days of curing compared to the control. This improvement was attributed to nano silica's high SiO₂ and lower CaO content, which promote hydration and pozzolanic reactions. It was found that nano silica played a crucial role for increasing the compressive strength of concrete. Nano silica's SiO₂ reacts with C₃A and C₃S, was accelerating the setting process and enhancing hydration product formation, leading to improved strength development. Additionally, nano silica's fine particles acted as fillers, reducing pore space, densifying the microstructure, and strengthening the concrete. Blending 30% surkhi with 2–4% nano silica yielded the highest strength improvements. Mix C_30‐4_ (30% surkhi and 4% nano silica) achieved the maximum compressive strength, exceeding the control by 35.4% at 28 days and 28.5% at 90 days of curing. In contrast, compressive strength of mix C_40‐2_ (40% surkhi and 2% nano silica) went down with a 16.1% reduction at 28 days and 14.28% at 90 days curing compared to the control mix. This reduction was likely due to slower pozzolanic reactions between the SiO₂ and Al₂O₃ in surkhi and the Ca(OH)₂ from cement hydration, which might require longer curing periods for full strength development.^[^ ^45^ ^]^ The hybridization of surkhi and nano silica was particularly beneficial. Surkhi, with its slight content of CaO concentration, lacks sufficient SiO₂ and Al₂O₃ for robust pozzolanic reactions. In contrast, nano silica provided abundant SiO₂ and adequate Al₂O₃.^[^ ^46^ ^]^ This combination leads to the production of abundant Ca(OH)₂ from nano silica and cement hydration, which reacted with the surplus SiO₂ and Al₂O₃ from surkhi and cement. The resulting pozzolanic reactions generate additional C‐S‐H, which densified the concrete microstructure and contributed to strength development. Also, the presence of M‐sand in the present concrete might be accelerated the compressive strength of concrete. The ternary combination of surkhi, nano silica and M‐sand might be increased the compressive strength of concrete. The cementitious paste and aggregate particles were better bonded by M‐sand's coarse, angular nature. Concrete's compressive strength increased as a result of this enhanced binding and greater particle packing brought about by regulated gradation.

### 6.6 Split Tensile Strength

**Figure 9:**
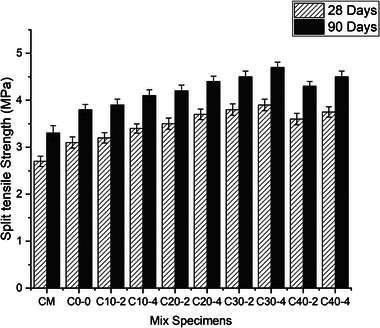
Split tensile strength of several concrete mixes at 28 days and 90 days of curing.

Split tensile strength tests were conducted on 300 mm‐long specimens with a diameter of 150 mm. These specimens underwent water curing and were tested using a two‐point loading method after 28 and 90 days, as shown in **Figure** **9**. The results indicated that the inclusion of surkhi and nano‐silica significantly influenced the split tensile strength of the concrete mix. The strength initially improved with increasing nano‐silica content in combination with surkhi, up to a surkhi replacement level of 30%. When metakaolin was replaced with 10% surkhi and 2% nano‐silica, the split tensile strength increased by 3.2% at 28 days and 2.6% at 90 days compared to the test results of traditional concrete. For samples with 30% surkhi and 2% nano‐silica, the split tensile strength rose to 3.8 N mm^2^ after 28 days and 4.5 N mm^2^ after 90 days, compared to 3.1 and 3.8 N mm^2^ for the nominal mix. The most notable improvement was observed in samples with 30% surkhi and 4% nano‐silica, which achieved split tensile strengths of 3.9 N mm^2^ at 28 days and 4.7 N mm^2^ at 90 days—the highest among all tested combinations. However, when the replacement percentage of metakaolin with surkhi increased to 40% with 2% nano‐silica, the split tensile strength dropped to 3.6 N mm^2^ at 28 days and 4.3 N mm^2^ at 90 days. Interestingly, with 4% nano‐silica at the same surkhi level, the strength slightly recovered to 3.75 N mm^2^ and 4.5 N mm^2^ at 28 and 90 days, respectively. These results suggest that while nano‐silica enhanced the tensile properties of concrete due to improved bonding and microstructure through the formation of calcium‐silicate‐hydrate (C‐S‐H) gel, excessive surkhi (beyond 30%) may increase porosity due to oversaturation, thereby compromising the bonding between cement particles. The addition of nano‐silica at higher levels, particularly at 4%, significantly enhances the split tensile strength of the concrete mix. This enhancement is most pronounced at 28 days, with strength gains diminishing over time but still remaining superior to conventional concrete mixes after 90 days. These findings underscore the optimal synergy between nano‐silica and surkhi at specific replacement levels for improving the tensile properties of concrete. M‐sand increased the split tensile strength of concrete because its rough and angular particles helped the surkhi and nano silica based cement matrix better interlock,. Compared to river sand, which had smoother and less sticky particles, this robust interlock resisted tensile stresses better.

### 6.7 Flexural Strength Factor for Different Concrete Mixes

The flexural strength results attained from the 28 and 90‐days flexural strength tests have been represented in terms of flexural strength factor as shown in **Table** **3**. Similarly, to the compression strength, it can be seen that the flexural strength of all samples improved with replacement of cement with metakaolin up to 30%. This was anticipated as an adequate curing and better packing density was provided. Based on the results obtained, the 28 days flexural strength increased as the surkhi content increased, which can be explained by the production of C–S–H from pozzolanic reactions.

**Table 3:** Flexural strength factor for different mixes of concrete.

Mix ID	Flexural strength factor		Average *k* value	Standard deviation
	28 Days	90 Days		
CM	1	1	1	0.000
C_0‐0_	1.08	1.12	1.1	0.028
C_10‐2_	1.15	1.26	1.205	0.078
C_10‐4_	1.19	1.31	1.25	0.085
C_20‐2_	1.24	1.36	1.3	0.085
C_20‐4_	1.28	1.39	1.335	0.078
C_30‐2_	2.15	2.37	2.26	0.156
C_30‐4_	2.32	2.38	2.56	0.170
C_40‐2_	1.23	1.41	1.32	0.127
C_40‐4_	1.27	1.46	1.365	0.134

Flexural strength factor (k) can be defined as a multiplication factor to obtain flexural strength of the binary combination of surkhi and nano silica replaced cement from traditional cement concrete. “k” values were obtained from Equation (2). Table 3 shows k values for different metakaolin replacements of LC3 cement for 28 and 90 days of curing.

(3) $$k=\frac{Flexural\;strength\;of\;metakaolin\;and\;nano\;silica\;based\;concrete}{Flexural\;strength\;of\;traditional\;concrete}$$

It was found that as the replacement of metakaolin with surkhi increased the average value of k for 28 days and 90 days curing also increased up to 30% replacement. The maximum average k value was noticed for the mix of C_30‐4_, which is 2.32 times of the traditional concrete at 28 days and 2.38 times at 90 days curing. The binary combination of surkhi and silica fume increased the internal packing density of concrete and achieved the maximum k value. The corresponding standard deviation also found the highest value among other mixes. Beyond 30% replacement of metakaolin with surkhi and gypsum with silica fume, both average k value and the standard deviation suddenly decreased. But when the amount of nano silica increased up to 4% with the combination of 40% surkhi, that reduction of average k value increased but there is no improvement in standard deviation value.

## 7 Conclusions

The present investigation demonstrated with a high degree of confidence that surkhi and nano‐silica could be effectively utilized as partial substitutes for metakaolin and gypsum, while M‐sand could fully replace natural sand in concrete production. These alternative building materials enhanced the strength and microstructural properties of concrete. For optimal performance at 28 and 90 days, cement substitution with up to 30% surkhi and 4% nano‐silica, along with 100% replacement of natural sand with M‐sand, is recommended. The following conclusions were drawn from the study:

- *Flowability*: The combination of surkhi and nano‐silica as metakaolin and gypsum replacement materials improved the flow capacity of LC3 concrete. However, metakaolin replacement should be restricted to 30% to maintain performance. The maximum recorded flow value was 525 mm for mix C_30‐4_, which was 1.64 times greater than traditional concrete. While M‐sand reduced workability, this reduction was compensated by incorporating nano‐silica.
- *Density and Microstructure*: Increased replacement of metakaolin and gypsum with the combination of surkhi and nano‐silica resulted in denser concrete compared to traditional mixes. However, when surkhi content exceeded 30%, voids were observed in SEM images. The fine particle size and high SiO₂ content of surkhi and nano‐silica facilitated the formation of ettringite and additional C‐S‐H gel during the hydration process, enhancing microstructural properties.
- *Nanoindentation Properties*: The modulus and hardness value for traditional concrete was lower compared to the newly developed concrete mixes. Mix C_30‐4_ exhibited a gradual increase in modulus, with average modulus and hardness values 3.1%–17.2% higher than those of traditional concrete.
- *Thermal Resistance*: Nano‐silica demonstrated superior resistance to mass loss at elevated temperatures compared to surkhi. Cement replacement with up to 30% surkhi showed excellent resistance to mass loss under high‐temperature conditions.
- *Mechanical Properties*: At 28 and 90 days of curing, the mechanical properties of concrete with 30% surkhi and 4% nano‐silica were significantly improved. This combination enhanced C‐S‐H gel production during hydration. Additionally, the use of M‐sand increased packing density, resulting in higher compressive and split tensile strengths compared to traditional concrete.
- *Flexural Strength Factor*: The flexural strength of the C_30‐4_ mix was 2.32 and 2.38 times higher than that of traditional concrete at 28 days and 90 days curing. The binary combination of surkhi and nano‐silica increased internal packing density and achieved the maximum k‐value, albeit with high standard deviation.

In summary, the binary blend of surkhi and nano‐silica, coupled with M‐sand as a complete replacement for natural sand, offers a sustainable and high‐performance alternative to traditional concrete.

## Conflict of Interest

The authors declare no conflict of interest.

## Data Availability

The data that support the findings of this study are available from the corresponding author upon reasonable request.

## References

[1] J. J. Biernacki , J. W. Bullard , G. Sant , K. Brown , F. P. Glasser , S. Jones , T. Ley , R. Livingston , L. Nicoleau , J. Olek , F. Sanchez , R. Shahsavari , P. E. Stutzman , K. Sobolev , T. Prater , J. Am. Ceram. Soc. 2017, 100, 2746. 28966345 10.1111/jace.14948 PMC5615410

[2] G. Habert , S. A. Miller , V. M. John , J. L. Provis , A. Favier , A. Horvath , K. L. Scrivener , Nat. Rev. Earth Environ. 2020, 1, 559.

[3] D. Xu , Y. Cui , H. Li , K. Yang , W. Xu , Y. Chen , Cem. Concr. Res. 2015, 78, 2.

[4] D. O. Oyejobi , A. A. Firoozi , D. B. Fernández , S. Avudaiappan , Results Eng. 2024, 24, 102846.

[5] S. Supino , O. Malandrino , M. Testa , D. Sica , J. Clean. Prod. 2016, 112, 430.

[6] G. Velmurugan , L. Natrayan , J. S. Chohan , P. Vasanthi , S. Angalaeswari , P. Pravin , S. Kaliappan , D. Arunkumar , Biomass Conv. Bioref. 2024, 14, 26011.

[7] D. Carran , J. Hughes , A. Leslie , C. Kennedy , Int. J. Archit. Herit. 2012, 6, 117.

[8] B. Lothenbach , K. Scrivener , R. D. Hooton , Cem. Concr. Res. 2011, 41, 1244.

[9] R. Snellings , P. Suraneni , J. Skibsted , Cem. Concr. Res. 2023, 171, 107199.

[10] M. C. G. Juenger , R. Snellings , S. A. Bernal , Cem. Concr. Res. 2019, 122, 257.

[11] R. Siddique , J. Klaus , Appl. Clay Sci. 2009, 43, 392.

[12] C.‐S. Poon , S. Azhar , M. Anson , Y.‐L. Wong , Cem. Concr. Compos. 2003, 25, 83.

[13] A. Nadeem , S. A. Memon , T. Y. Lo , Constr. Build. Mater. 2014, 62, 67.

[14] S. Wild , J. M. Khatib , A. Jones , Cem. Concr. Res. 1996, 26, 1537.

[15] J. Ding , Z. Li , ACI Mater. J. 2002, 99, 393.

[16] A. Kijjanon , T. Sumranwanich , S. Tangtermsirikul , Adv. Cem. Res. 2024, 37, 24.

[17] J. L. Xia , Z. Jiang , W. Zhang , F. Leng , J. Wang , X. Zhao , Constr. Build. Mater. 2024, 425, 136114.

[18] W. Bai , G. Suo , C. Yuan , J. Guan , C. Xie , L. Li , J. Build. Eng. 2024, 94, 109921.

[19] R. Badrinarayan , D. Shirish , R. Gangadhar , Indian J. Sci. Technol. 2016, 9, 1.

[20] M. Alamri , T. Ali , H. Ahmed , M. Z. Qureshi , A. Elmagarhe , M. Adil Khan , A. Ajwad , M. Sarmad Mahmood , Heliyon 2024, 10, 29014. 10.1016/j.heliyon.2024.e29014 PMC11021967 38633632

[21] J. H. A. Rocha , R. D. Toledo Filho , Case Stud. Constr. Mater. 2024, 21, 03996.

[22] C. Zhong , L. Zhang , W. Mao , S. Xing , J. Chen , J. Zhou , Case Stud. Constr. Mater. 2024, 20, 02978.

[23] P. P. A. , D. K. Nayak , B. Sangoju , R. Kumar , V. Kumar , Constr. Build. Mater. 2021, 278, 122347.

[24] A. M. Said , M. S. Zeidan , M. T. Bassuoni , Y. Tian , Constr. Build. Mater. 2012, 36, 838.

[25] K. Khan , W. Ahmad , M. N. Amin , S. Nazar , Nanomaterials 2022, 12, 1989. 35745327 10.3390/nano12121989 PMC9228660

[26] K. Behfarnia , N. Salemi , Constr. Build. Mater. 2013, 48, 580.

[27] B. B. Mukharjee , S. V. Barai , Constr. Build. Mater. 2014, 55, 29.

[28] M. Bastami , M. Baghbadrani , F. Aslani , Constr. Build. Mater. 2014, 68, 402.

[29] L. G. Li , J. Y. Zheng , J. Zhu , A. K. H. Kwan , Constr. Build. Mater. 2018, 168, 622.

[30] D. Suriya , S. P. Chandar , P. T. Ravichandran , Buildings 2023, 13, 1126.

[31] S. K. Rao , P. Sravana , T. C. Rao , Constr. Build. Mater. 2016, 122, 191.

[32] Radhakrishna , K. Praveen Kumar , Mater. Today Proc. 2018, 5, 25412.

[33] S. Ramkumar , R. Dineshkumar , Mater. Today Proc 2020, 21, 36.

[34] S. Suresh , J. Revathi , J. Comput. Theor. Nanosci. 2017, 14, 929.

[35] S. K. Rao , P. Sravana , T. C. Rao , Int. J. Pavement Res. Technol. 2016, 9, 289.

[36] P. Yi , H. Du , C. Chai , Y. Li , Y. Hu , X. Sun , W. Liu , F. Xing , J. Build. Eng. 2024, 98, 111335.

[37] M. Samani , Y. K. Ahlawat , A. Golchin , H. A. Alikhani , A. Baybordi , S. Mishra , Ö. Simsek , BMC Plant Biol. 2024, 24, 598. 38914950 10.1186/s12870-024-05311-1 PMC11197238

[38] Y. Zhao , Y. Zhang , Solids 2023, 4, 24.

[39] S. Sahith Reddy , M. Achyutha Kumar Reddy , IOP Conf. Ser. Earth Environ. Sci. 2021, 796, 012037.

[40] V. Nežerka , Z. Slížková , P. Tesárek , T. Plachý , D. Frankeová , V. Petráňová , Cem. Concr. Res. 2014, 64, 17.

[41] L. Natrayan , S. A. Kalam , S. Sheela , P. Paramasivam , K. Shanmugam , Discov. Appl. Sci. 2024, 6, 662.

[42] B. B. Mukharjee , S. V. Barai , Constr. Build. Mater. 2014, 71, 570.

[43] L. Sorelli , G. Constantinides , F.‐J. Ulm , F. Toutlemonde , Cem. Concr. Res. 2008, 38, 1447.

[44] A. A. Aliabdo , A.‐E. M. Abd‐Elmoaty , H. H. Hassan , Alex. Eng. J. 2014, 53, 151.

[45] C. Shi , Can. J. Civ. Eng. 2001, 28, 778.

[46] M. Sivaperumal , J. V. S. P. Kumar , L. Natrayan , S. Kaliappan , J Aust Ceram Soc 2025.

